# Total Breakthrough Strategy in Hydrophilic Interaction Chromatography for IP‐RPLC × HILIC Separation of Oligonucleotides

**DOI:** 10.1002/jssc.70388

**Published:** 2026-03-04

**Authors:** Megane K. Aebischer, Yusraa Abdurahman, Davy Guillarme

**Affiliations:** ^1^ School of Pharmaceutical Sciences University of Geneva Geneva Switzerland; ^2^ Institute of Pharmaceutical Sciences of Western Switzerland University of Geneva Geneva Switzerland

**Keywords:** HILIC, IP‐RPLC × HILIC, LC × LC, online comprehensive 2D‐LC, solvent mismatch, total breakthrough

## Abstract

Solvent mismatch between the two dimensions is one of the main limitations in two‐dimensional liquid chromatography (2D‐LC) and presents a significant challenge for method development. Although 2D‐LC provides a powerful means to increase peak capacity for oligonucleotides analysis compared to conventional one‐dimensional LC, solvent incompatibility remains a major obstacle that discourages broader development of such methods. In this work, we investigate a technical solution that can be easily implemented and that eliminates solvent mismatch effects during 2D‐LC analysis of oligonucleotides. This approach is based on the total breakthrough behavior of oligonucleotides, which is a phenomenon that allows the injection of large volumes into the second dimension (^2^D) without peak distortion. In this work, we showed that under appropriate conditions, oligonucleotides exhibit total breakthrough behavior in HILIC. This behavior in HILIC is particularly advantageous, as the IP‐RPLC × HILIC configuration offers improved mass spectrometry (MS) compatibility compared to IP‐RPLC or HILIC × IP‐RPLC. Assuming an IP‐RPLC × HILIC configuration, we systematically investigated the composition of first‐dimension (^1^D) fractions and the ^2^D‐HILIC parameters influencing total breakthrough to identify the key factors promoting this behavior. Our results offer clear guidance for implementing successful IP‐RPLC × HILIC conditions that avoid mismatch effects for oligonucleotides analysis while maintaining a high injection volume in the second dimension. This work demonstrates the potential of the total breakthrough strategy for implementing 2D‐LC methods with HILIC as the second dimension.

## 1 Introduction

As oligonucleotides (ONs) drug candidates progress toward clinical use, the demand for high‐resolution analytical approaches to enable comprehensive quality assessment continues to grow. While IP‐RPLC remains the benchmark for ON analysis [1], complementary modes, such as HILIC [2, 3] are increasingly used [4]. However, despite optimization efforts, full resolution of all impurities in a 1D‐LC method is still difficult to obtain.

To address these limitations, 2D‐LC is used for its superior resolution of closely related ON impurities [2, 3, 5, 6, 7, 8, 9]. However, comprehensive 2D‐LC typically requires large ^2^D‐injection volumes, which increases the risk of solvent incompatibility effects between dimensions. Indeed, mismatch between the ^1^D‐effluent and the initial ^2^D‐mobile phase can cause breakthrough effects, which are responsible for peak broadening, fronting, deformation, splitting, or partial elution near the column dead time due to limited interaction with the stationary phase. Conventional strategies to mitigate solvent mismatch exist, but often rely on complex instrumentation [10, 11].

A simple alternative exploits total breakthrough (TB) behavior of analytes in ^2^D, in which large injection volumes produce a non‐retained breakthrough peak followed by a sharp retained peak once a critical ^2^D‐injection volume is exceeded [12, 13]. This phenomenon allows large ^2^D‐injection volumes without compromising chromatographic performance. However, not all the compounds present this behavior. In a previous work, we demonstrated the TB behavior of ONs under IP‐RPLC conditions [14], enabling its use in 2D‐LC configurations with IP‐RPLC as ^2^D. However, although feasible, employing ion‐pairing reagents (IPR) in ^2^D hyphenated to the MS can lead to detector contamination with long term ionization suppression [15]. When HILIC is employed in ^2^D, it is directly coupled to the MS, while the IPR from the ^1^D‐IP‐RPLC are not transferred to the MS. This configuration therefore minimizes the risk of instrument contamination, in contrast to HILIC × IP‐RPLC systems where IP‐RPLC is directly connected to the MS.

In this context, the present work aims to demonstrate the occurrence of TB behavior for ONs in HILIC. A systematic investigation is carried out to evaluate the influence of key chromatographic parameters on TB behavior in an IP‐RPLC × HILIC configuration, with the objective of identifying conditions that promote TB during ON analysis.

## 2 Materials and Methods

### 2.1 Chemicals and Reagents

RNase‐free water, ammonium formate, ammonium acetate (≥97 %, ACS reagent), acetic acid (≥99 %), hexylamine (≥99 %, HA), and triethylamine (≥99.5 %, TEA) were obtained from Sigma‐Aldrich (Buchs, Switzerland). Milli‐Q water (Millipore, Bedford, MA) and LC‐MS grade acetonitrile (ACN) from Thermo Fisher Scientific (Reinach, Switzerland) were used for mobile‐phase preparation.

ONs samples were obtained from Eurogentec (Seraing, Belgium) and prepared as 100 µM stock solutions in RNase‐free water. These solutions were stored at −20°C until use. The study employed several thymidine homopolymers (dT10, dT15, dT20, dT25, dT35, and dT40) and two others 20‐mer sequences. One of these sequences was designed to remain in a linear structure (CTA‐CTA‐CTA‐GCT‐ACT‐ACG‐TA), while the other was expected to fold into a hairpin structure (ATG‐CAT‐GAT‐CGA‐TGA‐TCG‐TA).

### 2.2 Instrumentation

1D‐LC analyses were carried out using an ACQUITY UPLC H‐Class instrument (Waters, Milford, MA, USA) equipped with a binary solvent delivery pump, an auto‐sampler with flow‐through‐needle (FTN) injection. Each injection was performed using a 100 µL sample loop and injected volumes varied between 0.5 and 30 µL. The inner diameter of the tube between the column and the detector was 0.1 mm. UV detection was achieved with a UV photodiode array (PDA) detector with a 0.5 µL UV flow cell, monitoring the signal at 260 nm.

2D‐LC IP‐RPLC × HILIC experiments were performed on a 1290 Infinity II series 2D‐LC instrument from Agilent Technologies (Waldbronn, Germany). The instrument included a ^1^D 1290 binary pump with a 35 µL mixer, a ^2^D 1290 high‐pressure binary pump with a 35 µL mixer, a 1290 thermostated autosampler with a flow‐through needle injector equipped with a 20 µL sample loop, a ^1^D Multicolumn Thermostat (MCT) column compartment, a ^2^D Thermostated Column Compartment (TCC), a ^1^D Variable Wavelength Detector (VWD), and a ^2^D diode‐array UV absorbance detector (DAD). The interface connecting the two dimensions was a 4‐position/10‐port Active Solvent Modulation (ASM) valve connected via four 1.9 µL transfer capillaries (170 × 0.12 mm) to two 6‐position/14‐port parking deck valves, mounted with two sample loop of 120 µL each for LC × LC experiment. A pressure release kit (PRK) was installed to protect the ^1^D‐VWD flow cell from pressure spikes arising from the 2D‐LC valve switching.

### 2.3 Chromatographic Conditions

1D‐LC experiments were performed to simulate the injection conditions used in ^2^D‐HILIC separations. For each analysis, samples were prepared by diluting the 100 µM stock solutions to a final concentration of 2 µM in RNase‐free water (unless stated otherwise) or in the selected injection solvent. ONs were analyzed by HILIC on a Waters Premier BEH Amide column (50 × 2.1 mm, 1.7 µm, 130 Å; dead volume ≈ 105 µL) at 60°C and 0.7 mL/min. The mobile phase were prepared with A: Water + 25 mM ammonium acetate and B: Acetonitrile, and a linear gradient from 70% to 40% B over 1 min, followed by 2 min re‐equilibration was applied.

A one‐factor‐at‐a‐time (OFAT) strategy was applied to the dT20 sample, with the corresponding experimental variations summarized in Table 1. For each tested condition, the critical breakthrough and TB volumes (*V*_crit,BT_ and *V*_crit,TB,_ as described in Section 3.1) were determined by gradually increasing the injection volume in 1 µL increments until the onset of breakthrough and TB was observed.

**TABLE 1:** *V*_crit,B_ and *V*_crit,TB_ values obtained under various chromatographic conditions for a dT20 sample, along with a summary of strategies to decrease *V*_crit,TB_ and promote TB. The numbers in italics correspond to the line numbers associated which allow direct comparison of the impact of each parameter on *V*_crit,B_ and *V*_crit,TB_ (with the figures referenced in the manuscript). .

	^2^D HILIC conditions					^1^D IP‐RPLC collected fraction				
	Column length [mm] (0 vs. 1)	Column temperature [°C] (0 vs. 2)	Salt concentration [mM] (0 vs. 3, Figure 2)	Ammonium salt nature (0 vs. 4, Figure 2)	pH (0 vs. 5)	H_2_O (%) (0 vs. 6 vs. 7)	IPR nature (8 vs. 9, Fig. 3)	IPR concentration [mM] (6 vs. 9 vs. 10, Fig. 3)	*V*_crit,B_ measured ± 1 uL	*V*_crit,TB_ measured ± 1 uL
0	50	60	25	Acetate	6.7	100	—	0	3	10
1	150	60	25	Acetate	6.7	100	—	0	10	30
2	50	40	25	Acetate	6.7	100	—	0	4	14
3	50	60	50	Acetate	6.7	100	—	0	5	14
4	50	60	25	Formate	6.7	100	—	0	4	14
5	50	60	25	Acetate	5.1	100	—	0	4	16
6	50	60	25	Acetate	6.7	90	—	0	4	20
7	50	60	25	Acetate	6.7	50	—	0	14	22
8	50	60	25	Acetate	6.7	90	HA	10	4	15
9	50	60	25	Acetate	6.7	90	TEA	10	4	16
10	50	60	25	Acetate	6.7	90	TEA	50	4	12
Strategies to decrease *V*_crit,TB_ and promote TB	Decrease column length	Increase temperature	Decrease salt concentration	Decrease salt polarity	Increase pH	Increase H2O %	Increase IPR hydrophobicity	Increase IPR concentration		

2D‐LC IP‐RPLC × HILIC conditions were performed using conditions described in Table 2.

**TABLE 2:** Comprehensive 2D‐LC (IP‐RPLC × HILIC) conditions used to demonstrate the applicability of the total breakthrough strategy.

First dimension (^1^D)	
**Sample nature**	Mixture of a dT10, dT15, dT20, dT30, dT35, and dT40 in Rnase free water
**Sample concentration (µM)**	14
**Injection volume (uL)**	10
**Solvent A**	50 mM TEA and 50 mM acetic acid in water
**Solvent B**	50 mM TEA and 50 mM acetic acid in ACN
**Initial composition (%B)**	7
**Final composition (%B)**	14.5
**Gradient time (min)**	60
**Flow rate (µL/min)**	50
**Temperature (°C)**	80°C
**Column**	BEH C18 130A, 2.1 × 150 mm, 1.7 µm
**Detection**	UV 260 nm, 80Hz

Second dimension (^2^D)	
**Injection volume (uL)**	75.3
**Sampling time (min)**	1.5
**Solvent A**	50 mM ammonium acetate in water pH 6.7
**Solvent B**	ACN
**Initial composition (%B)**	70
**Final composition (%B)**	40
**Gradient time (min)**	1
**Equilibration time (min)**	0.5
**Flow rate (µL/min)**	1500
**Temperature (°C)**	80°C
**Column**	Premier BEH Amide 130A, 50 × 2.1, 1.7 µm
**Detection**	UV 260 nm, 80Hz

### 2.4 Data Analysis

1D data acquisition and instrument control were managed using Empower Pro 3 (Waters). 2D Data acquisition was performed using MassHunter WorkStation Data Acquisition software (Agilent). 2D‐LC data visualization was carried out using the *Visualization* module of the online 2D‐Smart Calculator (freely available) [16].

## 3 Results and Discussion

### 3.1 Total breakthrough Behavior of ONs Under HILIC Conditions

**FIGURE 1:**
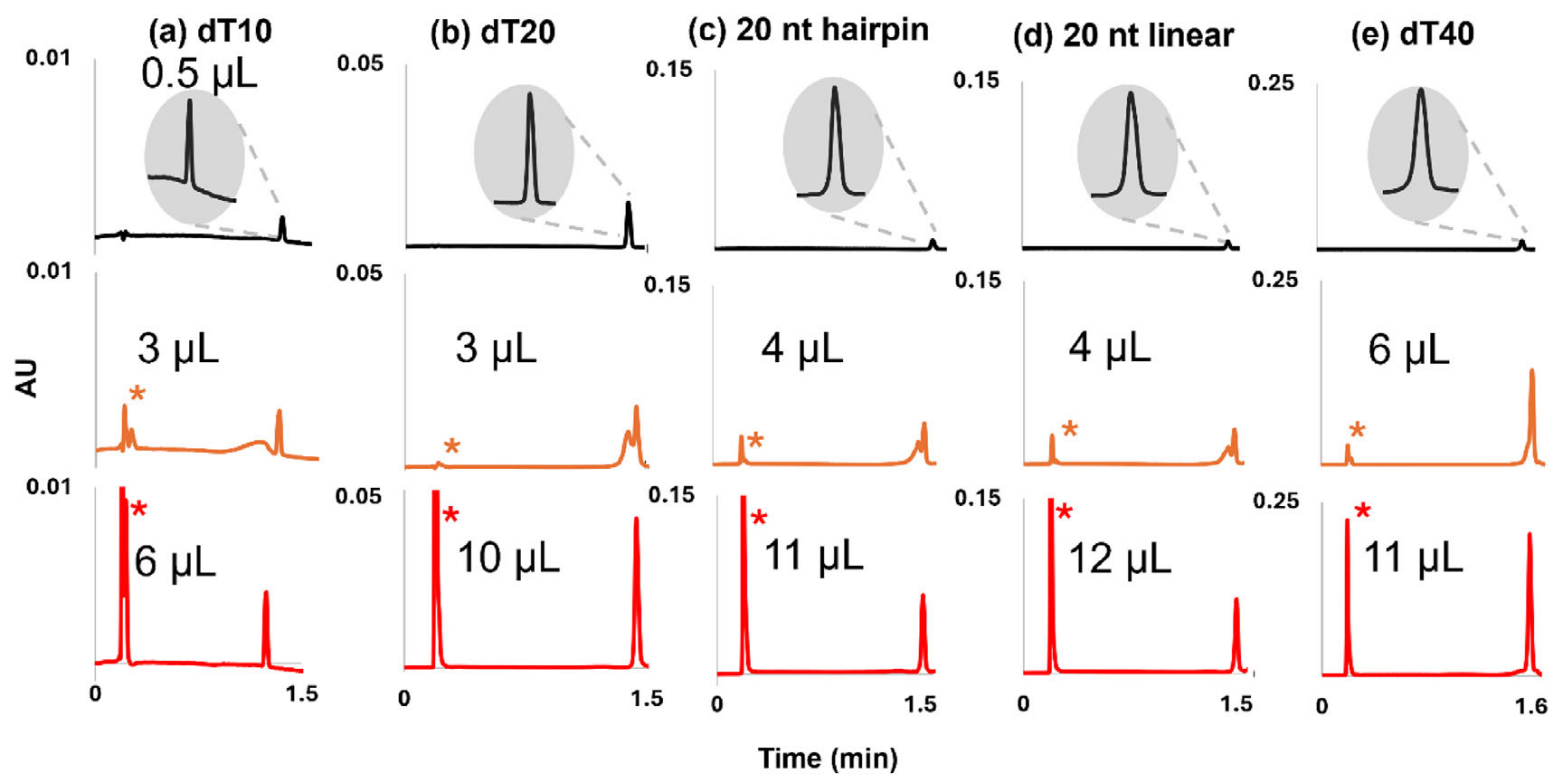
Effect of oligonucleotide length and sequence on breakthrough behavior under gradient elution: (a) dT10, (b) dT20, (c) 20‐mer hairpin, (d) 20‐mer linear, and (e) dT40 (2 µM) analyzed on a Premier BEH amide column (50 × 2.1 mm, 1.7 µm); flow rate 0.7 mL/min; column temperature 60°C; 70%–40% B; 1‐minute gradient. Mobile phases: A, 25 mM ammonium acetate in water; B, ACN. Injection solvent: H_2_O; detection at 260 nm. The asterisk marks the non‐retained breakthrough peak. Black trace represents the reference peak with 0.5 µL injected, orange trace corresponds to V_crit,B_, and red trace indicates V_crit,TB_.

The TB behavior in HILIC mode for various ONs is presented in Figure 1. Several ONs were studied, differing in length and sequence (dT10, dT20, dT40, as well as 20‐mer sequences with linear or hairpin configuration).

In HILIC, ONs display TB behavior, similarly to what was reported in IP‐RPLC [14]. At small injection volumes (0.5 µL, < 1% of *V*_0_; black trace, Figure 1), the chromatogram displays a single, symmetrical retained peak. This signal, referred to here as the “reference peak,” reflects conditions under which the injected plug is too small to induce injection‐related disturbances. As the injected volume increases, a point is reached at which a breakthrough signal first emerges; this volume is defined as the critical breakthrough volume, *V*_crit,B_.

Upon further increasing the injection volume, TB behavior becomes evident for all ONs (red trace, Figure 1). Under these conditions, the chromatogram is characterized by three features: (i) A sharp retained peak with a width similar to that of the reference peak, (ii) the absence of intermediate peak, and (iii) a well‐defined breakthrough peak eluting near the column dead volume. The injection volume at which this pattern appears is denoted *V*_crit,TB_.

For ONs analyzed under IP‐RPLC conditions—and similarly for small molecules and peptides in RPLC systems that also display TB behavior—*V*_crit,TB_ is typically about 1.4 times larger than *V*_crit,B_. This relationship [12, 14] can be expressed as:

(1) $$V_{\mathrm{crit},\mathrm{TB}}\approx 1.4*V_{\mathrm{crit},B}=1.4*k_{s}*V_{0}$$

where *V*_0_​ is the column dead volume and *k_s_* is the retention factor in the injection solvent.

Contrary to these observations, ONs show no fixed proportionality in HILIC: The *V*_crit,TB_ /*V*_crit,B_​ ratio ranges from 1.8 to 3.0 depending on the selected ON (Figure 1). As there was no proportionality, both values *V*_crit,B_ and *V*_crit,TB_ were always experimentally measured in this study.

Even if a clear relationship between *V*_crit,B_ and *V*_crit,TB_ was not identified, both values increase with ON length and hydrophobicity. For example, more hydrophobic sequences (dT20 in Figure 1b) display lower *V*_crit,B_ and *V*_crit,TB_ values (respectively 3 and 10 µL) than less hydrophobic 20‐mers (Figure 1d) (respectively 4 and 12 µL). This trend likely arises from the lower *k_s_* associated with the elution of shorter and less hydrophobic ONs. Indeed, shorter and less hydrophobic sequences offer more polar interactions with the stationary phase, which are responsible for the retention in HILIC. ONs with stronger retention are thus more likely to form a retained peak rather than being eluted as breakthrough, resulting in increased *V*_crit,B_ and *V*_crit,TB_. These results demonstrate that, under HILIC conditions, TB behavior occurs for all ONs, but *V*_crit,TB_ depends on the ON analyzed.

According to previous studies [14], the on‐off retention mechanism, associated with high *S* values (the slope of the LSS retention model) [17], was hypothesized to play a key role in determining whether a molecule can exhibit TB behavior. This assumption is supported by earlier RPLC and HILIC investigations on small molecules and peptides [12], which possess low *S* values. In those studies, negatively charged compounds with low *S* values did not show TB behavior in either RPLC or HILIC, but instead displayed peak distortion or partial breakthrough, even at high injection volumes [12]. Conversely, positively charged compounds with similarly low *S* values were found to exhibit TB behavior, highlighting the influence of molecular charge on the occurrence of TB.

However, when working in IP‐RPLC with ONs (17), which are negatively charged compounds with high *S* values [18], TB behavior occurs. Our hypothesis is that at low *S* values (typical of small peptides and small molecules), the ability to TB behavior is primarily governed by the charge. However, at higher *S* values (>15), the influence of charge seems to decrease and the on–off retention mechanism becomes predominant. Thus, the high *S* value emerges as the main parameter governing TB behavior, independent of molecular charge. For instance, 20‐mers ONs exhibit relatively high *S* values in HILIC (≈ 16–19 for dT20 under standard conditions [17]). They remain negatively charged under HILIC conditions but still show clear TB behavior (Figure 1).

### 3.2 Factors Influencing Total Breakthrough in IP‐RPLC × HILIC

The TB behavior of ONs in HILIC can be exploited to mitigate issues related to solvent‐strength mismatch when coupling a ^1^D mode with HILIC in ^2^D, such as in IP‐RPLC × HILIC. In this configuration, breakthrough behavior depends on (i) ON properties (as discussed in Section 3.1), (ii) ^2^D‐HILIC parameters, and (iii) ^1^D‐IP‐RPLC conditions that define the ^2^D‐injection solvent. To elucidate the mechanism and identify the optimal conditions that promote TB, we systematically investigated the factors governing breakthrough. To simulate ^2^D‐HILIC, 1D HILIC experiments were conducted using an OFAT approach, by screening key variables to identify those minimizing *V*_crit,B_ and *V*_crit,TB_ (Table 1).

#### 3.2.1 Influence of ^2^D‐HILIC Conditions on Breakthrough

This subsection aims to identify HILIC conditions that can be applied in a 2D‐LC system to enhance the occurrence of TB. Several HILIC conditions were therefore evaluated.

Based on our previous findings [14], gradient conditions do not affect *V*_crit, B_ and *V*_crit, TB_ because these parameters are independent of *k_s_* (Equation 1). Experimental observations confirmed that *V*_crit, B_ and *V*_crit, TB_ remained unchanged across different gradient durations and flow rates. In addition, for a constant *k_s_*, both volumes increase linearly with *V*_0_ (Equation 1). This trend is further supported by the results shown in Table 1: Reducing the column length (from 150 to 50 mm) while maintaining the same internal diameter and stationary‐phase chemistry leads to a decrease in both *V*_crit, B_ and *V*_crit, TB_ by the same factor.

To promote TB without modifying column dimensions, alternative HILIC conditions were explored. As illustrated in Table 1, higher temperatures reduce both *V*_(_crit, B_)_ and *V*_(_crit, TB_)_ relative to lower temperatures. This behavior can be attributed to temperature‐induced changes in *k_s_*. Increasing the temperature weakens the interactions between the ONs and the stationary phase, thereby facilitating earlier elution of the analyte and enhancing breakthrough.

**FIGURE 2:**
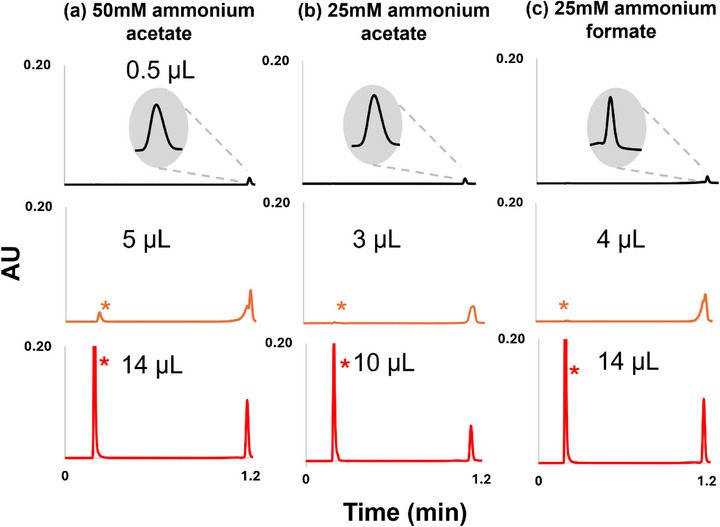
Influence of salt type and concentration in the mobile phase on breakthrough behavior during gradient elution for dT20 (2 µM): (a) 50 mM ammonium acetate, (b) 25 mM ammonium acetate, and (c) 25 mM ammonium formate in mobile phase A. Column: Premier BEH amide (50 × 2.1 mm, 1.7 µm); flow rate 0.7 mL/min; 70%–40% B; 1‐minute gradient. Mobile phases: A, water containing the indicated salt; B, ACN. Injection solvent: H_2_O; detection at 260 nm. The asterisk denotes the non‐retained breakthrough peak. Black trace represents the reference peak with 0.5 µL injected, orange trace corresponds to *V*_crit,B_, and red trace indicates *V*_crit,TB_.

As shown in Figure 2 and Table 1, both the type and salt concentration strongly influence *V*_crit, B_ and *V*_crit, TB_ in HILIC. Indeed, the retention factor in injection solvent *k_s_* depends on the surface chemistry of the stationary phase. Altering the salt modifies this surface: At high salt proportion in the mobile phase (50 mM ammonium acetate, Figure 2a), the larger number of NH_4_^+^ ions effectively neutralize residual silanols, reducing electrostatic repulsion with the anionic ONs, which increases *V*_crit,TB_. In contrast, at lower salt level (25 mM ammonium acetate, Figure 2b), fewer NH_4_^+^ ions mask the silanols, strengthening electrostatic repulsion, promoting TB at smaller injection volumes. Salt nature exerts a similar effect. *V*_crit,B_ and *V*_crit,TB_ were higher with ammonium formate compared to ammonium acetate (Figure 2b,c). Because acetate (CH_3_COO^−^) is less polar and a stronger H‐bond acceptor than formate (HCOO^−^), acetate more effectively competes for analyte H‐bond donors, weakens ON‐column interactions, and reduces HILIC retention. Thus, ammonium acetate favors TB at lower *V*_crit,TB_. Accordingly, when the goal is to induce TB, working with low concentrations of ammonium acetate in the mobile phase is advised.

The last parameter investigated regarding HILIC conditions was the mobile phase pH. Higher pH values enhance silanols ionization, promote electrostatic repulsion, and thus favor TB. Consequently, both V_crit,B​_ and V_crit,TB​_ decrease at higher pH, as illustrated in Table 1.

#### 3.2.2 ^1^D‐IP‐RPLC Conditions Affecting Breakthrough in ^2^D‐HILIC

Breakthrough behavior also depends on the ^1^D‐conditions. Indeed, the composition of the ^2^D‐injected fraction is determined by the ^1^D‐elution composition. As the ^2^D‐injected fraction composition impacts *k_s_* (Equation 1), understanding the injection parameters that influence breakthrough behavior is essential for identifying the ^1^D conditions that promote TB.

To this purpose, under the assumption of IP‐RPLC conditions in ^1^D, a systematic investigation was performed to assess how factors related to the collected fraction affect the breakthrough behavior of ONs in HILIC.

As previously reported [14], the amount of water present in the injected fraction plays a major role in governing breakthrough. Water acts as a strong eluent in HILIC; therefore, lowering its proportion in the injected plug leads to higher values of *V*_crit,B​_ and *V*_crit,TB_ (Table 1). Increasing the water content weakens the interaction between the analyte and the stationary phase during the injection step, allowing the analyte to travel with the solvent front and thus favoring TB. Among the investigated parameters, the composition of the injection solvent showed the greatest influence on *V*_crit,TB_: Decreasing H_2_O from 100 % to 50 % in the injected fraction increases the *V*_crit,TB_ value by more than 2‐fold. Therefore, when applying a TB strategy in IP‐RPLC × HILIC, maintaining high H_2_O content in the collected fraction is advisable.

**FIGURE 3:**
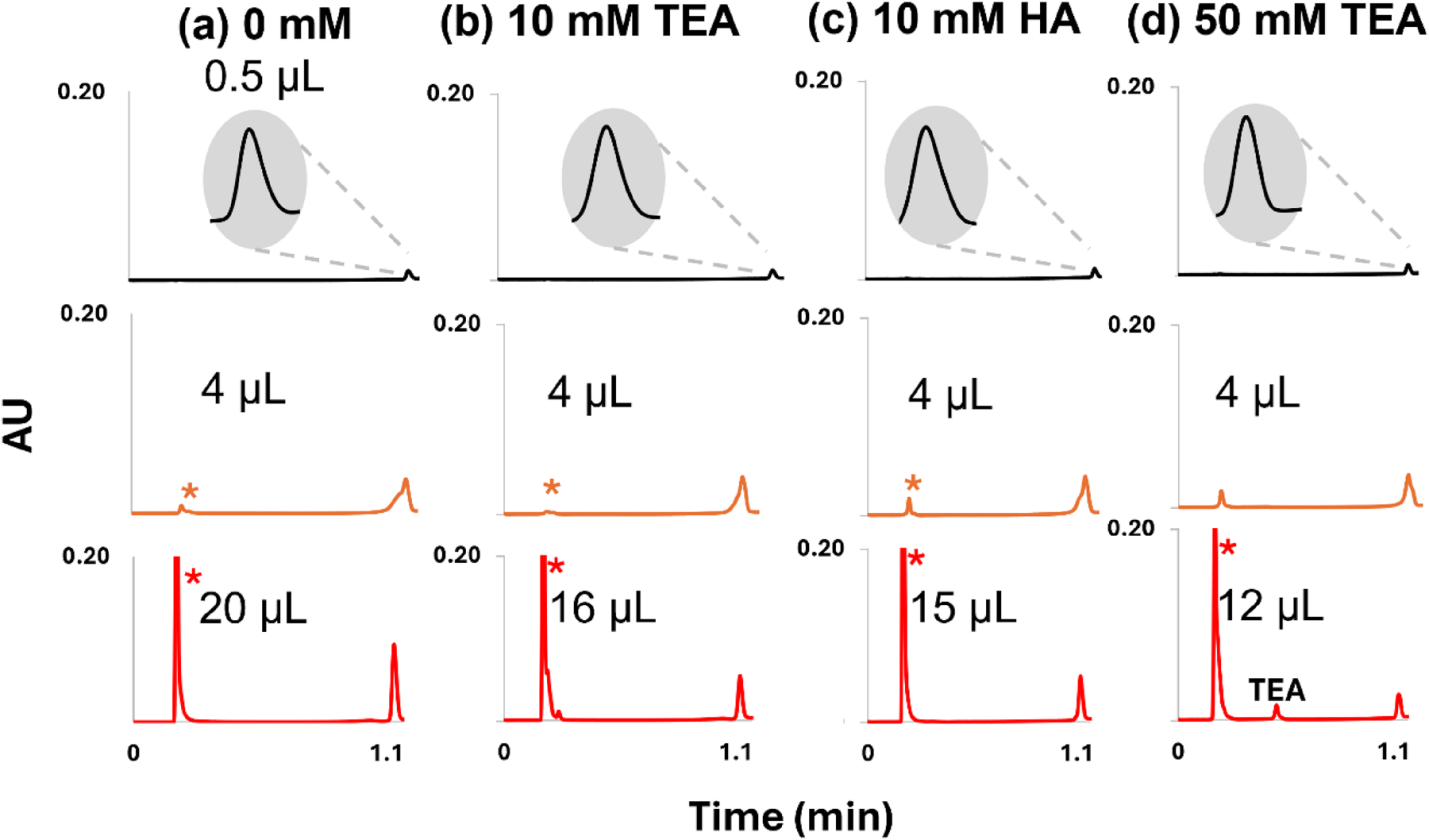
Effect of ion‐pair reagent type and concentration in the injected fraction on breakthrough behavior for dT20 (2 µM) under gradient elution: (a) 0 mM, (b) 10 mM TEA, (c) 10 mM HA, and (d) 50 mM TEA. Column: Premier BEH amide (50 × 2.1 mm, 1.7 µm); flow rate 0.7 mL/min; 70%–40% B; 1‐minute gradient. Mobile phases: A, 25 mM ammonium acetate in water; B, ACN. Injection solvent: H_2_O/ACN 90:10; detection at 260 nm. The asterisk marks the non‐retained breakthrough peak. Black trace represents the reference peak with 0.5 µL injected, orange trace corresponds to *V*_crit,B_, and red trace indicates *V*_crit,TB_.

To adjust the H_2_O content of the ^1^D‐IP‐RPLC effluent, the type and concentration of IPR used in ^1^D‐IP‐RPLC must be chosen carefully. In standard IP‐RPLC of ONs, the mobile phase typically contains 10–50 mM IPR, whose hydrophobicity may vary, to provide the ion pairing necessary for retaining the negatively charged ONs on a C18 column. In this study, a slight decrease in *V*_crit,TB_ was observed when using HA instead of TEA as the IPR (Table 1, Figure 3b,c). The higher hydrophobicity of HA reduces ON retention in HILIC [19], increasing the propensity of breakthrough and thus lowering *V*_crit,TB_. However, the effect is rather minor resulting in similar breakthrough threshold. Furthermore, it is important to note that the use of HA also induces a lower proportion of H_2_O in the ^1^D‐collected fraction. Indeed, in IP‐RPLC, a more hydrophobic IPR increases retention, which in turn requires a higher elution strength (organic solvent proportion) for elution. The fact that HA and TEA appear to promote similar *V*_crit,TB_ is true only when comparing fractions collected at the same H_2_O proportion. In a real 2D‐LC setup with IP‐RP in ^1^D, TEA is more favorable for promoting TB, not because of its direct presence in the collected fraction, but because it results in a higher H_2_O content in the collected fraction, thereby enhancing TB. For this reason, TEA is recommended to promote TB, and higher TEA concentrations further enhance this effect. As shown in Figure 3a,b,d and Table 1, *V*_crit, TB_ ​decreases as the IPR concentration in the injected fraction increases.

### 3.3 Application of the Total Breakthrough Strategy in an IP‐RPLC × HILIC Setup

**FIGURE 4:**
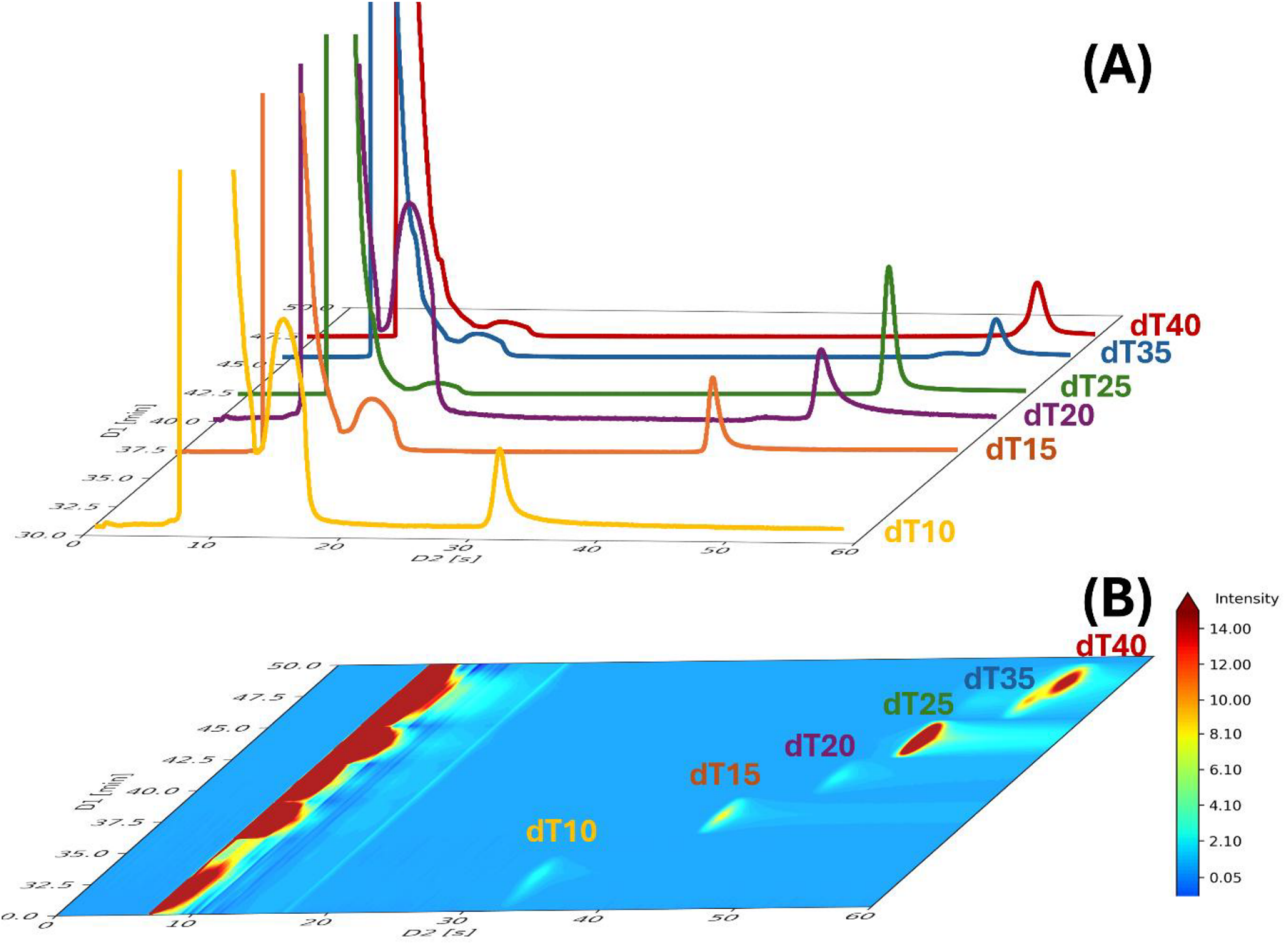
Comprehensive IP‐RPLC × HILIC analysis of an ON ladder under conditions promoting total breakthrough. (A) Individual second‐dimension chromatograms obtained for each ON fraction. (B) Corresponding 2D contour plot.

As shown in Figure 4, the optimized conditions promoting TB were successfully implemented in a comprehensive IP‐RPLC × HILIC setup. A simple ONs ladder mixture (dT10–dT40) was analyzed under the conditions reported in Table 2 to demonstrate the applicability of the TB strategy.

Despite the high ^2^D‐injection volume (75.3 µL, ∼72% of the ^2^D‐HILIC column volume), peak shapes remained unaffected. Although a breakthrough peak was observed for each ON (Figure 4A,B), separation performance was not compromised.

## 4 Conclusion

This work complements a previous study and extends the understanding of the TB behavior of ONs in HILIC. We demonstrated for the first time that ONs exhibit TB in HILIC, independently of their size, sequence, or secondary structure. Exploiting this behavior provides a simple and effective way to increase the ^2^D‐injection volume when using HILIC as the second dimension, ensuring full MS compatibility.

A systematic evaluation of IP‐RPLC × HILIC conditions identified the key factors that govern TB in HILIC. The data show that the most effective conditions to decrease *V*_crit,TB_ and thus to promote TB are: (i) increase of H_2_O content in the injected fraction by working with IPR with low hydrophobicity in the ^1^D‐IP‐RPLC, (ii) increase IPR concentration in the ^1^D‐IP‐RPLC, limiting ON‐HILIC stationary phase interaction, (iii) lower ammonium‐salt concentration in ^2^D‐HILIC mobile phases or use of the less polar (acetate instead of formate), to increase electrostatic repulsion, (iv) higher column temperature, which decreases H‐bonded interactions between ON and the stationary phase, (v) shorter column length, which increases the injection volume/column volume ratio, (vi) increase pH to increase electrostatic repulsion and promote TB.

These findings demonstrate the potential of this strategy for 2D‐LC methods using HILIC as the second dimension and offer clear guidance for implementing successful IP‐RPLC × HILIC that take advantage of the TB strategy separations for reliable ON analysis.

## Author Contributions

**Megane Aebischer**: conceptualization, date curation, investigation, methodology, validation, writing ‐ original draft **Yusraa Abdurahman**: Formal Analysis, data curation, investigation, methodology, writing ‐ review and editing. **Davy Guillarme**: Writing review and editing; Supervision; project Administration; Funding Acquisition, resources.
